# MiRNA Dysregulation in Brain Injury: An *In Silico* Study to Clarify the Role of a MiRNA Set

**DOI:** 10.2174/1570159X22666240808124427

**Published:** 2024-08-08

**Authors:** Francesco Sessa, Cristoforo Pomara, Flavia Schembari, Massimiliano Esposito, Emanuele Capasso, Mauro Pesaresi, Eduardo Osuna, Efehan Ulas, Christian Zammit, Monica Salerno

**Affiliations:** 1 Department of Medical, Surgical and Advanced Technologies “G.F. Ingrassia”, University of Catania, Catania, Italy;; 2 Faculty of Medicine and Surgery, “Kore” University of Enna, 94100 Enna, Italy;; 3 Department of Advanced Biomedical Science-Legal Medicine Section, University of Naples “Federico II”, 80131 Naples, Italy;; 4 Section of Legal Medicine, Department of Biomedical Sciences and Public Health, Polytechnic University of Marche, Via Tronto, Ancona, 60126, Italy;; 5 Department of Forensic Medicine. University of Murcia. 30120 Murcia, Spain;; 6 Faculty of Medicine, Department of Biostatistics and Medical Informatics, Kirklareli University, Kirklareli, Turkey;; 7 Department of Anatomy, Faculty of Medicine and Surgery, University of Malta, Msida 2080, Malta

**Keywords:** Brain injury, biomarkers, miRNA, diagnosis, prognosis, theranomiRNA

## Abstract

**Background:**

The identification of specific circulating miRNAs has been proposed as a valuable tool for elucidating the pathophysiology of brain damage or injury and predicting patient outcomes.

**Objective:**

This study aims to apply several bioinformatic tools in order to clarify miRNA interactions with potential genes involved in brain injury, emphasizing the need of using a computational approach to determine the most likely correlations between miRNAs and target genes. Specifically, this study centers on elucidating the roles of miR-34b, miR-34c, miR-135a, miR-200c, and miR-451a.

**Methods:**

After a careful evaluation of different software available (analyzing the strengths and limitations), we applied three tools, one to perform an analysis of the validated targets (miRTarBase), and two to evaluate functional annotations (miRBase and TAM 2.0).

**Results:**

Research findings indicate elevated levels of miR-135a and miR-34b in patients with traumatic brain injury (TBI) within the first day post-injury, while miR-200c and miR-34c were found to be upregulated after 7 days. Moreover, miR-451a and miR-135a were found overexpressed in the serum, while miRNAs 34b, 34c, and 200c, had lower serum levels at baseline post brain injury.

**Conclusion:**

This study emphasizes the use of computational methods in determining the most likely relationships between miRNAs and target genes by investigating several bioinformatic techniques to elucidate miRNA interactions with potential genes. Specifically, this study focuses on the functions of miR-34b, miR-34c, miR-135a, miR-200c, and miR-451a, providing an up-to-date overview and suggesting future research directions for identifying theranomiRNAs related to brain injury, both at the tissue and serum levels.

## 1 INTRODUCTION

Brain injury is a multifaceted and often devastating condition that can occur as a result of various events, including trauma, stroke, infection, and neurodegenerative diseases. Such events are highly disabling for the patient and have a heavy impact on the economic evaluations of public health interventions. Therefore, identifying the underlying molecular mechanisms involved in brain injury is an important area of research. A major challenge is that numerous cellular processes are involved. In recent years, experimental research has turned special attention toward microRNAs (miRNAs), small noncoding RNA molecules that regulate gene expression at the post-transcriptional level. Notably, they are among the most frequently investigated emerging players in the pathophysiology of brain injury [1-3].

miRNAs are short, single-stranded RNA sequences that are about 18-25 nucleotides long and are stable even in post-mortem samples. They are involved in several crucial physiological processes, including development, cell proliferation, differentiation, and apoptosis. miRNAs function primarily by binding to complementary sequences in the 3' untranslated regions (UTRs) of messenger RNA (mRNA) molecules, leading to degradation or translational repression of the target mRNA. Through their ability to control the expression of several genes, miRNAs are essential for preserving cellular homeostasis. All these features make them ideal molecular biomarkers for both diagnosis and treatment of different diseases. However, to date, their applicability appears to be limited due to the necessity for translational experiments aimed at validating these biomarkers [4-6].

Emerging evidence suggests that miRNA dysregulation contributes to the pathogenesis of both acute and chronic brain injury. In cases of acute brain injury, such as traumatic brain injury (TBI), stroke, or brain hemorrhage, the rapid release of inflammatory factors triggers a cascade of molecular events that lead to secondary brain damage. These secondary injury mechanisms, including excitotoxicity, oxidative stress, neuroinflammation, and apoptosis, can further exacerbate the initial insult. Studies have revealed that miRNAs are actively involved in modulating these processes, either by serving as mediators or by directly regulating key signaling pathways [7-10].

Several miRNAs have been implicated in the pathophysiology of brain injury. For instance, miR-21, miR-146a, and miR-155 are upregulated in response to brain injury and are known to modulate inflammatory pathways by targeting various components of the immune response [11, 12]. MiR-181 family members have been shown to regulate neuronal apoptosis and synaptic plasticity, whereas miR-124 is involved in the regulation of neuronal differentiation. Dysregulation of these miRNAs can disrupt the delicate balance between pro-survival and pro-apoptotic signals in injured neurons, leading to the progression of injury [13-15].

In addition to acute brain injuries, miRNA dysregulation has also been implicated in chronic neurodegenerative conditions, including Alzheimer's disease (AD), Parkinson's disease (PD), and amyotrophic lateral sclerosis (ALS) [16, 17]. These devastating disorders are characterized by progressive neuronal loss and cognitive decline, and mounting evidence suggests that miRNAs play a significant role in their pathogenesis. Dysregulated miRNAs have been observed in the brains of AD patients and have been found to influence the processing of amyloid precursor protein (APP) and the generation of β-amyloid plaques, a hallmark of the disease. Similarly, several miRNAs have been identified as key players in the aberrant protein aggregation and dopaminergic neuron degeneration seen in PD [16-18].

The comprehension of the functional implications of miRNA dysregulation in each organ is crucial, with particular significance to brain injury. Furthermore, the characterization of dysregulated miRNAs is not solely used to discern early indicators of injury but also to improvehe exploration of prospective therapeutic interventions through the silencing of these aberrant miRNAs [19, 20]. MiRNAs represent an attractive target for therapeutic intervention because of their ability to regulate multiple genes simultaneously. Modulation of specific miRNAs can potentially alter the expression of entire signaling networks, providing a powerful tool to manipulate disease progression. Indeed, several preclinical studies demonstrated promising results using miRNA-based therapies in various neurodegenerative disorders [21, 22].

According to the existing literature, five miRNAs have been frequently investigated concerning brain injury: 34b, 34c, 135a, 200c, and 451a. Studies have shown that patients with TBI had elevated levels of miRNAs 135a and 34b within the initial 24 hours post-injury, whilst miR-200c and miR-34c were overexpressed seven days after brain damage. In addition, miR-451a and miR-135a were found to be upregulated in the serum, while miRNAs 34b, 34c, and 200c demonstrated decreased serum levels post-brain injury [23, 24].

In this context, studies conducted *in silico* may be highly helpful in clarifying different molecular features and pointing out potential directions for future *in vivo* research. In order to clarify miRNA interactions with potential genes, this study aims to investigate several bioinformatic tools, emphasizing the need to use a computational approach to determine the most likely correlations between miRNAs and target genes. Specifically, this study centers on elucidating the roles of miR-34b, miR-34c, miR-135a, miR-200c, and miR-451a, aiming to present a comprehensive contemporary analysis of their functionality while proposing future research trajectories for the identification of theranomiRNAs related to brain damage, spanning both tissue and serum levels.

## 2 MATERIALS AND METHODS

In this study, we utilize *in silico* tools to examine the roles of various miRNAs, drawing from a recent literature review that highlighted the most extensively studied miRNAs in human models of brain injury.

### 2.1 Selection of miRNAs and *in silico* Tools

We performed a full review of the literature, focusing on the most promising miRNAs related to brain injury, both in animal and human studies. Based on our revision, this in silico study focused on the role of hsa-miR-34b, miR-34c, miR-135a, miR-200c, and miR-451a.

Today, numerous tools are available to perform *in silico* analysis. Based on a recent publication [25], after a careful evaluation of each application (analysis of the strengths and limitations), we decided to use three tools: one to perform an analysis of the validated targets (miRTarBase) and two to evaluate functional annotations (miRBase and TAM 2.0).

### 2.2 miRBase Tool

Accessible at https://www.mirbase.org (accessed on November 16, 2023), miRbase is an extensive database that enables users to look for published miRNA sequences and annotations. There are 38,589 items in version 22.1 that was utilized for this article. With every miRNA record in the miRBase database, there is a predicted sequence database that represents a miRNA transcript's hairpin segment. This offers important details on the exact position and composition of the mature miRNA sequence, or miR. For every miRNA in this investigation, we were able to get both the associated “Word cloud” image and its miRNA sequence. Additionally, the miRBase database includes a link to the top 100 articles retrieved from PubMed. From this list, we selected the top 5 articles based on their relevance and specifically focused on those investigating cerebral function. It is worth noting that the unique functionality of this tool of linking to PubMed articles has been thoroughly described by Kozomara *et al*. [26].

### 2.3 TAM 2.0 Tool

We employed the TAM 2.0 program to examine the functional annotation of the chosen miRNAs. This tool, which can be accessed for free by academics (accessed on November 16, 2023) at http://www.lirmed.com/tam2/, is based on an analysis of approximately 9k papers and allows the association of each miRNA set with various diseases, miRNA families, and transcription factors, and biological functions.

In summary, the main functionality of this tool includes the ability to upload a list of miRNAs and choose between overrepresentation and underrepresentation. Additionally, it allows for the selection of upregulation (or disease promotion) or downregulation (or disease suppression).

When entering mature miRNA names such as hsa-miR-34b, the tool converts them into the corresponding miRNA gene (hsa-mir-34b) based on its data setting. Alternatively, the program will examine all duplicate miRNA genes if the precise name of a duplicated miRNA gene is not given.

Through the utilization of this tool, we acquired comprehensive insights about the analyzed miRNA set, encompassing cell functions, associations with transcriptional factors, and their relevance to human diseases.

### 2.4 miRTarBase Tool

miRTarBase is a database that provides verified data on interactions between miRNAs and their target genes through biological experiments. The information in this database undergoes periodic revisions, and for this study, we utilized Release 9.0 beta (dated 15 September 2021, available at https://miRTarBase.cuhk.edu.cn/, accessed on 16 November 2023). This release includes data from 13,398 articles spanning 37 different species. It encompasses a total of 27,172 target genes and 4,630 miRNAs, resulting in 2,200,449 miRNA-target interactions. Harnessing this valuable resource, each miRNA under examination was analyzed to identify its target genes and explore the functional implications through gene ontology.

## 3 RESULTS

### 3.1 miRTarBase Tool Analysis

The first step was the analysis of the sequence for each miRNA (Table **1**).

This step is crucial to enable the computational analysis to be repeated.

**Fig. (1):**
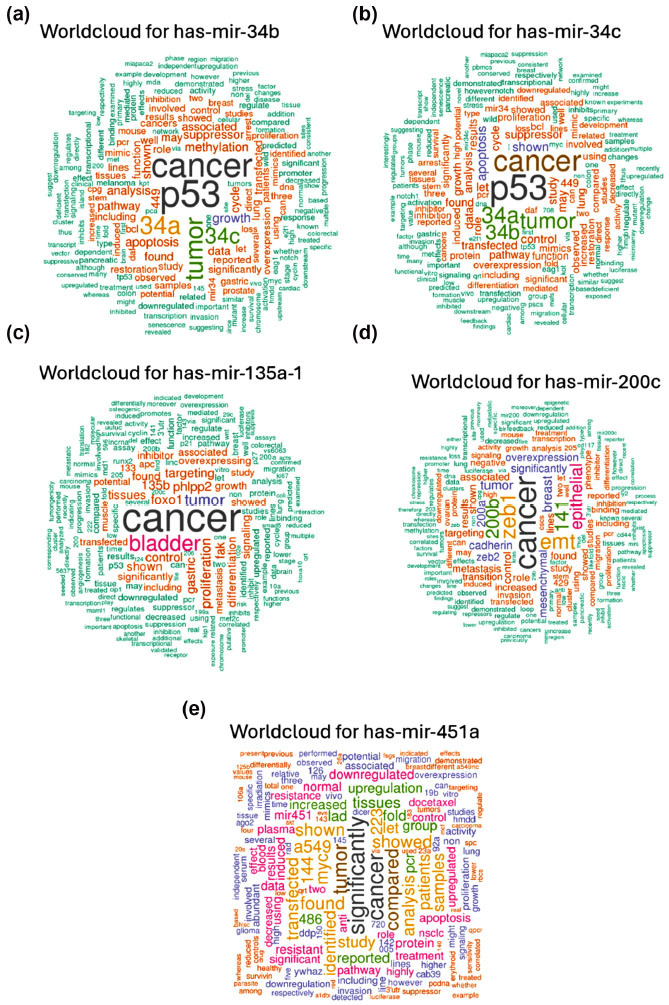
Word cloud for hsa-mir-34b (**a**); hsa-miR-34c (**b**); hsa-miR-135a-1 (**c**); hsa-miR-200c (**d**); hsa-miR-451a (**e**).

With the use of this technology, we were able to generate the word clouds (Fig. **1**) for each miRNA under investigation.

Based on these graphic representations, hsa-miR-34b, miR-34c, miR-135a, and miR-200c are more frequently related to the out-of-topic keywords, given that “cancer” or “p53” was more frequent than “brain”, while miR-451a matched with “significantly” and “dysregulation”. Nevertheless, in each word cloud, there are numerous keywords that may be associated with apoptosis or cellular death. Thus, these miRNAs could be involved in regulating cell life post-brain injury, which is deemed crucial in the context of damage or injury.

Furthermore, to explore the implication of these miRNAs in brain injury, we employed the same tool to scrutinize the top 100 articles associated with each miRNA, subsequently selecting and summarizing the top 5 articles pertinent to the research theme, as presented in Table **2**. For each article, we focused on the following details: its ranking (indicating its position among the 100 articles listed in the database), primary author, publication year, title, the number of sentences that reported the name of the selected miRNA, and the additional human miRNAs investigated (if only the miRNA of interest was investigated, then it is reported by the symbol “/“).

Upon analyzing the output, the results summarized in Table **2** indicate a high score for hsa-miR-135a-1 and hsa-miR-451a, whereas the other miRNAs exhibit a lower correspondence as mediators of brain injury.

These results are in line with the word cloud, demonstrating that the function of these miRNAs is only partially involved in brain injury regulation.

### 3.2 Functional Annotation Analysis *via* the TAM 2.0 Tool

In the left panel of the TAM 2.0 tool, we inserted our miRNA set that was found to be overexpressed in tissue samples: hsa-mir-135a, hsa-mir-34b, hsa-mir-34c, and hsa-mir-200c.

Upon analyzing the results related to the tissue specificity category, this set of miRNAs appears to be specific for brain development and aging. Moreover, these miRNAs are involved in specific brain pathologies such as PD and AD. In this way, it is possible that these miRNAs as involved as brain injury mediators. The link between cell activities and the uploaded miRNA collection is summarized in Fig. (**2**).

In Fig. (**3**), we have summarized the gene ontology (GO) related to brain development.

This miRNA set seems to be related to the following specific functions: induction of apoptosis by p53, apoptosis signaling, commitment to apoptosis, induction of apoptosis, caspase-dependent programmed cell death, apoptotic cell death, apoptotic programming, apoptosis activator activity, activation of apoptosis, cellular suicide, type I programmed cell death, apoptosis, cell suicide, signaling (initiator) caspase activity, programmed cell death by apoptosis, and apoptotic Following this, we conducted an analysis of the miRNA set utilizing the Comparison Wizard tool, wherein hsa-mir-135a and hsa-mir-451a were designated as up-regulated miRNAs, while hsa-mir-34b, hsa-mir-34c, and hsa-mir-200c were categorized as under-regulated miRNAs, based on insights obtained from the literature review. As summarized in Table **1**, examination of brain tissue expression revealed that hsa-mir-135a and hsa-mir-451a were up-regulated immediately after brain injury, while hsa-mir-34b, hsa-mir-34c, and hsa-mir-200c were found to be under-regulated 7 days post-injury. Conversely, all miRNAs seemed to be upregulated in serum samples.

As summarized in Fig. (**4**), these miRNAs are implicated in various brain neurodegenerative diseases, such as PD and AD. Consequently, these miRNAs could play a pivotal role in mediating cellular damage.

### 3.3 miRTarBase Tool: Report about Experimentally Validated miRNA-target Interactions of the Selected miRNAs

We utilized the miRTarBase program to determine the interaction between our miRNA collection and their respective target.

The first analysis focuses on hsa-mir-34b-5p interactions that have been empirically confirmed. Fig. (**5**) summarizes the key findings about the interactions between this miRNA and the gene targets in human research based on the tool's results.

**Fig. (6):**
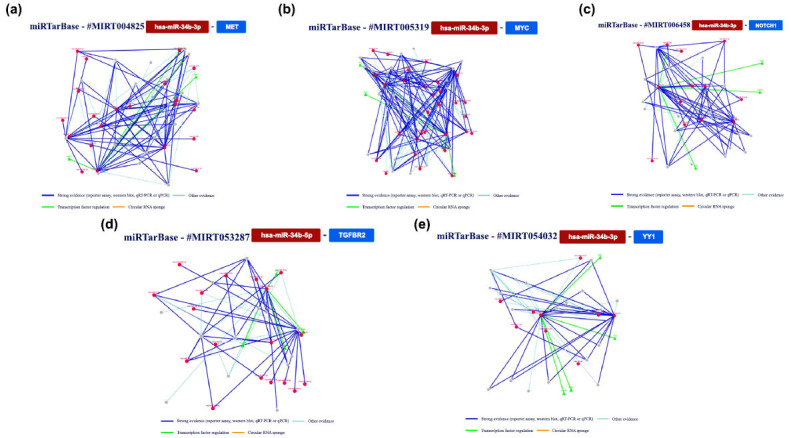
The regulatory network of has-miR-34b and the MET gene (**a** and **b**) the regulatory network of interactions between the tested miRNA and *MYC* gene; (**c**) the regulatory network of interaction between miR-34b and *NOTCH1* gene; the regulatory network of has-miR-34b and *TGFBR2* gene (**d**) and *YY1* gene (**e**).

Additionally, we examined the relationship again using five other approaches. In particular, we examined how the gene targets *MET*, *MYC*, and *NOTCH1* interacted with the network, transforming growth factor beta receptor 2 (*TGFBR2)* and *YY1* (Fig. **6**).

The *MET* gene encodes a protein belonging to the receptor tyrosine kinase family and the product of the proto-oncogene *MET*. Human cancer is caused by changes in the *MET* gene. Numerous malignancies have been linked to amplification and subsequent overexpression, which renders the receptor's function independent of *HGF*. Autosomal recessive deafness and Type 1 Papillary Renal Cell Carcinoma are diseases linked to *MET*. Apoptotic pathways are among the *MET*-related pathways; their participation may be crucial in the body's reaction to brain damage.

The nuclear phosphoprotein that the proto-oncogene *MYC* gene encodes is involved in apoptosis, cellular transformation, and the progress of the cell cycle. Together with the associated transcription factor *MAX*, the encoded protein forms a heterodimer. This complex controls the transcription of particular target genes by binding to the consensus sequence found in the E-box DNA. This gene is commonly amplified in a variety of human malignancies. Translocations affecting this gene have been linked to multiple myeloma and Burkitt lymphoma in human patients. The self-renewal of embryonic stem cells is regulated by somatic reprogramming (by similarity).

One of the proteins in the NOTCH family is encoded by the *NOTCH1* gene. The structural features of this family of Type I transmembrane proteins include many repetitions of the epidermal growth factor-like (*EGF*) in the extracellular domain and multiple distinct domain types in the intracellular domain. Processes linked to cell fate specification, differentiation, proliferation, and survival require the Notch signaling system. This gene, which acts as a receptor for membrane-bound ligands, is linked to aortic valve disease, Adams-Oliver syndrome, T-cell acute lymphoblastic leukemia, chronic lymphocytic leukemia, and head and neck squamous cell carcinoma. It is involved in the differentiation of Bergmann glia and represses both neuronal and myogenic differentiation throughout cerebellar development. It also serves as a receptor for neuronal DNER. It is likely to be involved in some part of cell specification and/or differentiation during post-implantation development. Additionally, it could have a role in neurogenesis, somite formation, and mesoderm development. Removing HIF1AN from HIF1A could improve HIF1A's function. This is necessary for the regulation of protective astrogenesis from the subventricular zone (SVZ) niche following damage by *THBS4*.

The transmembrane protein encoded by *TGFBR2* contains a protein kinase domain, binds to *TGF-beta*, and forms a heterodimeric complex with the *TGF-beta* receptor type-1. This receptor/ligand complex phosphorylates proteins, which then reach the nucleus and control the transcription of genes linked to cancer, wound healing, immunosuppression, cell cycle arrest, and proliferation of cells. This gene has been linked to the development of many kinds of cancers, Loeys-Deitz aortic aneurysm syndrome and Marfan syndrome. The same receptor is responsible for transducing the *TGFB2* signal from the cell surface into the cytoplasm, which in turn controls a wide range of physiological and pathological processes such as wound healing, the production of extracellular matrix, immunosuppression, carcinogenesis, and cell cycle arrest in epithelial and hematopoietic cells.

The protein-coding gene *YY1* Transcription Factor is a widely distributed transcription factor that is a member of the zinc finger protein class known as GLI-Kruppel. *YY1*-related diseases include insulinoma and Gabriele-De Vries Syndrome. *ESR*-mediated signaling and gene expression (transcription) are two of its associated mechanisms. Such pathways play an important role in development and differentiation and are involved in DNA repair.

Subsequently, we scrutinized the findings concerning the analysis of hsa-miR-34c. Through this examination, several gene targets were identified within human models. The principal outcomes of this analysis are consolidated and presented in Fig. (**7**).

Moreover, we conducted additional analysis on gene interactions that had been validated by at least five methods. Specifically, we examined the interaction network involving miR-34c and *E2F3*, *CDK4* (cyclin-dependent kinase 4), *MAZ,* and *MYC* genes. The main results are summarized in Fig. (**8**).

A transcription factor belonging to a small family that operates by means of a particular interaction with partner proteins is encoded by the *E2F3* gene. The encoded protein interacts directly with the retinoblastoma protein (pRB) to control the expression of genes related to the cell cycle by recognizing a particular sequence motif in DNA. Retinoblastoma and bilateral retinoblastoma are diseases linked to *E2F3*.

The retinoblastoma gene product *(Rb*) is known to be phosphorylated by the protein that the *CD4K* gene encodes. This protein is a member of the Ser/Thr protein kinase family. Several malignancies have been linked to mutations in this gene, which also affects the related proteins D-type cyclins, p16 (*INK4a*), and *Rb*. Key participants in the advancement of the cell cycle include *CDK4* and its companion *CDK6*. Research and development efforts are currently underway to find a cure for this complex, which has been linked to several forms of cancer. *MAZ* facilitates the activity of transcription factors that bind DNA, as well as the particular and cis-regulatory region sequence-specific DNA binding of RNA polymerase II. Furthermore, it plays a role in a number of activities, such as transcription by RNA polymerase II, signal transduction regulation, and gene expression regulation. Diseases associated with *MAZ* include Amyloidosis and Von Hippel-Lindau Syndrome.

By analyzing the hsa-miR-135a molecular interaction with this tool focusing on papers involving human models, we obtained the results summarized in Fig. (**9**).

Moreover, we conducted additional analysis on the interaction between hsa-miR-135a and the *JAK2* (considering that interaction with the *MYC* gene has been analyzed in the first paragraph), which was confirmed by four methods. This interaction network is illustrated in Fig. (**10**). As depicted in the figure, this network is easily compared with others, suggesting that its relationship seems clear.

The *JAK2* gene encodes a non-receptor tyrosine kinase that plays a central role in cytokine and growth factor signaling. Many inflammatory diseases and cancers are linked to mutations in this gene. The pleiotropic cytokine *IL6*, which is generated by B cells, T cells, dendritic cells, and macrophages to induce inflammation or an immunological response, is known to target this gene downstream. When a myeloproliferative disease is diagnosed, *JAK2* is one of the first diagnostic indicators to be examined. A class of tyrosine kinases known as *JAK*s (or Janus kinases) is connected to cytokine receptors. The *JAK-STAT* signaling pathway is started when *JAKs* phosphorylate the transcription factors known as *STATs* in response to receptor activation.

Using this tool to analyze an hsa-miR-200c model based on published papers focusing on human studies, we obtained the results summarized in Fig. (**11**).

Additionally, we further analyzed the interaction confirmed with at least five methods, exploring the interaction networks involving miR-200c and *ZEB1* gene, *TUBB3* gene, *BMI1* gene, *ZEB2* gene and *FN1* gene. The main results are summarized in Fig. (**12**).

The *ZEB1* gene encodes a zinc finger transcription factor. The encoded protein likely plays a role in the transcriptional repression of interleukin 2. Mutations in this gene have been associated with posterior polymorphous corneal dystrophy-3 and late-onset Fuchs endothelial corneal dystrophy. Moreover, it may play a role in positively regulating neuronal differentiation and repressing RCOR1 transcription activation during neurogenesis. This gene functions by repressing transcription through binding to the E box (5'-CANNTG-3'), and it promotes tumorigenicity by suppressing stemness-inhibiting miRNAs.

*TUBB3* encodes a member of the beta tubulin protein family classified as class III. Beta tubulins, comprising one of the two fundamental protein families alongside alpha tubulins, heterodimerize and assemble to construct microtubules. Primarily expressed in neurons, this protein likely participates in neurogenesis, axon guidance, and maintenance processes. Mutations within this gene are implicated in congenital fibrosis of the extraocular muscles type 3. Additionally, alternative splicing gives rise to multiple transcript variants. *TUBB3* is linked to diseases such as cortical dysplasia, which is complex, with other brain malformations 1. Additionally, the dorsal root ganglion axon's projection towards the spinal cord is influenced by this gene.

One of the main components of the polycomb group complex 1 (*PRC1*) is the ring finger protein encoded by the *BMI1* (*BMI1* proto-oncogene, polycomb ring finger) gene. This complex serves as a crucial epigenetic repressor of many regulatory genes involved in somatic stem cell self-renewal and embryonic development through chromatin remodeling. Additionally, this protein is essential for the repair of DNA damage. This gene is an oncogene whose abnormal expression has been linked to resistance to specific chemotherapies as well as a number of malignancies. Leukemia and mantle cell lymphoma are two diseases linked to *BMI1*. The *ZEB2* gene encodes a protein that belongs to the Zfh1 family of zinc finger/homeodomain proteins, which has two hands. It is a transcriptional repressor that binds to DNA and interacts with activated *SMADs*; it is found in the nucleus. Nervous system disorders and Mowat-Wilson Syndrome are diseases linked to *ZEB2*. This gene may control *TGF*-beta receptor signaling in skeletal dysplasias and promote the transformation of epithelial cells into mesenchymal cells in colorectal cancer, among other related pathways. Fibronectin is a glycoprotein that is expressed in the extracellular matrix and cell surface as well as in soluble dimeric or multimeric forms in plasma. The *FN1* gene codes for this protein. Spondylometaphyseal dysplasia, corner fracture type, and glomerulopathy with fibronectin deposits are diseases linked to *FN1*. The integrin route and the signaling downstream of *RAS* mutants are two of its related pathways.

Lastly, we examined and verified the interactions with hsa-miR-451a by experimentation. Fig. (**13**) summarizes the key findings about the interactions between this miRNA and the gene targets in human research based on the tool's results.

We conducted a more detailed analysis of the interactions between the miRNA and the respective genes, focusing specifically on those confirmed by a minimum of 5 validation methods. Consequently, we examined the interaction networks involving miR-451a and the genes migration inhibitory factor (*MIF)* (validated by 6 methods) and *CAB39* (validated by 5 methods), alongside the *IL6R* gene. The principal outcomes of this analysis are succinctly outlined in Fig. (**14**).

A lymphokine implicated in inflammation, immunoregulation, and cell-mediated immunity is encoded by the *MIF* gene. Through the inhibition of glucocorticoid anti-inflammatory actions, it contributes to the control of macrophage activity in host defense. Rheumatoid arthritis, systemic juvenile idiopathic arthritis, and systemic-onset juvenile idiopathic arthritis are among the illnesses linked to *MIF.* The innate immune system and interleukin-12 family signaling are two pathways that are frequently linked to this gene. These pathways regulate several cellular processes, including differentiation, proliferation, and apoptosis, as well as multiple pathological events. Protein serine/threonine kinase activator and kinase binding activity are enabled by the *CAB39* gene. This gene is involved in positive control of protein phosphorylation, peptidyl-serine phosphorylation, and intracellular signal transmission. Moreover, it may be implicated in hepatocellular carcinoma. A component of the interleukin 6 *(IL6)* receptor complex is encoded by the *IL6R* gene. Strong pleiotropic cytokine *IL6* controls the proliferation and differentiation of cells and is crucial for the immuneresponse. Numerous illnesses, including multiple myeloma, autoimmune disorders, and prostate cancer, are thought to be caused by the dysregulated production of *IL6* and its receptor. Furthermore, it plays a protective role during liver injury, as it is required for the maintenance of tissue regeneration. ‘Trans-signaling' in the central nervous system regulates energy and glucose homeostasis.

## 4 DISCUSSION

### 4.1 miR-34b and Target Genes

miR-34b is a miRNA that plays a crucial role in regulating the expression of the *MET* gene, which encodes the *MET* receptor tyrosine kinase [49]. The *MET* gene is involved in various cellular processes, including cell proliferation, survival, migration, and differentiation. Dysregulation of the *MET* gene has been implicated in several diseases, including cancer and neurological disorders [50]. In the context of brain injury, miR-34b has been shown to modulate the expression of the *MET* gene and influence neuronal survival and function. Studies have demonstrated that miR-34b targets the *MET* mRNA, leading to its degradation and inhibition. This downregulation of miR-34b is associated with increased *MET* protein levels, which in turn can contribute to the pathogenesis of brain injury. One possible mechanism through which miR-34b and *MET* gene dysregulation may contribute to brain injury is through the promotion of neuroinflammation [49]. Neuroinflammation represents a multifaceted phenomenon characterized by the activation of immune cells within the brain, culminating in the release of pro-inflammatory molecules. Studies have unveiled that the activation of the *MET* signaling pathway can amplify neuroinflammatory responses. Consequently, the downregulation of miR-34b and the ensuing upregulation of *MET* expression may exacerbate neuroinflammation, thereby precipitating neuronal damage and dysfunction [51, 52]. The role of miR-34b in the regulation of the *MYC* gene in brain injury is an area of ongoing research [49]. Dysregulation of the miR-34b/MYC axis has been implicated in tumorigenesis, tumor progression, and therapeutic resistance. One of the mechanisms by which miR-34b regulates the *MYC* gene is through direct binding and inhibition of its expression. miRNAs can bind to the mRNA molecules of target genes, leading to their degradation or inhibition of translation [16, 53]. In the case of the *MYC* gene, miR-34b can potentially target its mRNA and suppress its expression, thereby modulating its downstream effects. The downregulation of miR-34b has been associated with increased *MYC* protein levels, which can contribute to the pathogenesis of brain injury. *MYC* is a proto-oncogene that plays a crucial role in cellular proliferation, differentiation, and apoptosis. Dysregulation of *MYC* has been implicated in various diseases, including cancer and neurological disorders [54, 55]. Therefore, the dysregulation of miR-34b and the subsequent upregulation of *MYC* could potentially disrupt normal cellular processes and contribute to the development and progression of brain injury. Furthermore, the dysregulation of the miR-34b/MYC axis may also influence neuroinflammatory responses. Activation of the *MYC* signaling pathway has been shown to enhance neuroinflammation [56]. In the context of brain injury, this neuroinflammatory process can further exacerbate the injury and contribute to the pathogenesis of the condition. Consequently, the dysregulation of miR-34b and its effects on *MYC* expression may have repercussions on the inflammatory response following brain injury. Despite extensive research on the miR-34b/MYC axis across various diseases, including cancer, the specific role of miR-34b in brain injury and its therapeutic implications are still being explored. Further research is required to comprehensively understand the intricacies of miR-34b-mediated regulation of the *MYC* gene in the context of brain injury and neurological disorders [57]. This understanding could potentially lead to the development of targeted therapeutic strategies aimed at modulating the miR-34b/MYC axis to alleviate the effects of brain injury. The involvement of miR-34b in brain injury extends beyond its interactions with the *MET* and *MYC* genes. Emerging research suggests that miR-34b also plays a crucial role in regulating the *NOTCH1* gene, a key player in various cellular processes. *NOTCH1* is a transmembrane receptor that plays a vital role in diverse physiological and pathological processes, including brain development, neuronal differentiation, and cell fate determination. Dysregulation of *NOTCH1* signaling has been implicated in several neurological disorders, including brain injury [58, 59]. Studies have shown that miR-34b can directly target and suppress *NOTCH1* expression, disrupting normal cellular processes and contributing to the pathogenesis of brain damage. In the context of brain damage, the dysregulation of miR-34b and *NOTCH1* may exacerbate neuroinflammation and neuronal apoptosis and impair neurogenesis [60]. Increased *NOTCH1* expression has been associated with enhanced pro-inflammatory responses and impaired neuronal survival and regeneration. Targeting the miR-34b/NOTCH1 axis emerges as a promising therapeutic strategy for mitigating brain injury, aiming to restore miR-34b levels or inhibit *NOTCH1* expression to attenuate neuroinflammatory responses, promote neuronal survival, and enhance neurogenesis [61]. However, further research is needed to fully elucidate the intricate regulatory mechanisms and downstream effects of the miR-34b/NOTCH1 axis in brain injury, facilitating the development of novel therapeutic interventions to alleviate its detrimental effects on brain injury and improve patient outcomes. While the specific role of miR-34b in regulating the *TGFBR2* gene in brain injury remains unclear, extensive studies on miR-34b in cancer and other diseases provide valuable insights. MiR-34b, a tumor suppressor miRNA, plays a crucial role in regulating various genes involved in cellular processes, such as *MET, MYC*, and *NOTCH1*, which are implicated in brain injury. In the case of brain injury, the potential role of miR-34b in regulating *TGFBR2* remains unclear [62]. However, *TGFBR2* is known to be involved in various cellular functions, including cell growth, differentiation, and apoptosis, which are crucial in brain development and repair processes. Dysregulation of *TGFBR2* signaling is known to contribute to neuroinflammation, neuronal damage, and impaired neurogenesis, all leading to brain injury [63]. Therefore, understanding the role of miR-34b in regulating *TGFBR2* in the context of brain damage could provide valuable insights into potential therapeutic targets for mitigating brain injury. Further studies are warranted to elucidate the specific interactions between miR-34b and *TGFBR2* in brain injury, potentially leading to the development of targeted therapies for brain injury and related neurological disorders. In the context of brain injury, the dysregulation of miR-34b and its impact on *YY1* (a ubiquitous transcription factor that plays a crucial role in development) expression may influence the inflammatory response. *YY1* is a negative regulator of p53, a tumor suppressor protein involved in various cellular processes, including apoptosis and DNA repair. By targeting *YY1*, miR-34b may influence the activities of p53 and its downstream signaling pathways, potentially affecting the inflammatory response in brain injury [64]. Dysregulation of p53 signaling has been implicated in neuroinflammation and neuronal damage. Furthermore, miR-34b has been shown to play a role in regulating cancer stem cells (CSCs) and reversing epithelial-mesenchymal transition (EMT) [65]. Although the specific role of miR-34b on *YY1* in brain injury is not well understood, targeting the miR-34b/YY1 axis may have therapeutic implications for attenuating neuroinflammatory responses, promoting neuronal survival, and enhancing neurogenesis. Further research is needed to fully comprehend the interactions between miR-34b and *YY1* in brain injury and develop targeted therapies that can modulate the miR-34b/YY1 axis for potential therapeutic interventions.

### 4.2 miR-34c and Target Genes

miR-34c, a member of the miR-34 family, has been extensively investigated in cancer research as a tumor suppressor gene. However, its role in brain injury and in the regulation of the *E2F transcription factor 3 (E2F3)* gene remains poorly understood. The *E2F3* is an oncogene that has been implicated in various cancers and is associated with poor prognosis [66]. Recent research has revealed intricate interactions between *E2F3* and miRNAs, including miR-34c. Evidence indicates that miR-34c can target and reduce the expression of the E2F3 protein in different cellular contexts. This suggests that miR-34c may potentially play a role in regulating *E2F3* expression in the brain and, thus, influence the pathogenesis of brain injury [67]. The downregulation of *E2F3* by miR-34c could have therapeutic implications for mitigating brain injury, as *E2F3* is known to be involved in cell cycle progression, proliferation, and apoptosis. However, it is important to note that the role of miR-34c on *E2F3* in brain injury has not been extensively studied [68]. Further research is necessary to elucidate the specific regulatory mechanisms and downstream effects of miR-34c on *E2F3* in brain injury. Understanding these molecular interactions could potentially lead to the development of targeted therapeutic strategies to alleviate the detrimental effects of brain injury and improve patient outcomes.

miR-34c has also been found to target and regulate the expression of *CDK4*, a crucial protein involved in cell cycle regulation and cell proliferation. *CDK4* plays a critical role in controlling the progression of the cell cycle by forming a complex with cyclin D. This complex phosphorylates pRB, leading to the release of *E2F* transcription factors and subsequent cell cycle progression [69]. Dysregulation of *CDK4* has been associated with various diseases, including cancer and neurodegenerative disorders. Studies have shown that miR-34c directly targets the 3' UTR of *CDK4* mRNA, leading to its downregulation, resulting in cell cycle arrest and inhibition of cell proliferation, exacerbating neuronal injury [67]. By targeting *CDK4*, miR-34c could serve as a key regulator in mitigating the detrimental effects of brain injury. However, the exact mechanisms underlying miR-34c-mediated regulation of *CDK4* following brain injury are still not fully understood and require further investigation [70]. Unraveling the intricate interactions between miR-34c and *CDK4* in brain injury could provide valuable insights into the development of targeted therapeutic strategies aimed at modulating *CDK4* expression and cell cycle progression. Ultimately, such interventions may foster neuroprotection and facilitate recovery in individuals affected by brain injury.

The specific role of miR-34c on the *MAZ* gene in brain injury is not well documented. Further research is needed to elucidate the potential interactions between miR-34c and the *MAZ* gene in the context of brain damage. Overall, miR-34c plays a crucial role in regulating cellular processes involved in brain injury, and further investigation into its interactions with specific genes, such as *MAZ,* could provide valuable insights into the underlying mechanisms and potential therapeutic targets [71].

Although miR-34c has been extensively investigated within the realm of cancer, particularly concerning its regulatory function on the *MYC* proto-oncogene, its precise impact on the *MYC* gene in the context of brain damage remains insufficiently documented. Within the realm of brain damage, understanding miR-34c’s role in regulating *MYC* could be of significant interest. MiR-34c is recognized in its pivotal role in modulating cellular processes involved in brain damage, including apoptosis, inflammation, and oxidative stress [66, 72].

Gaining insight into the potential interactions between miR-34c and the *MYC* gene in the context of brain damage could provide a valuable understanding of the underlying mechanisms involved and might suggest potential new therapeutic targets. By targeting *MYC*, miR-34c may help modulate pathways involved in neuroprotection or neuroregeneration. Nonetheless, additional research is required to elucidate the specific role of miR-34c on the *MYC* gene in brain damage and to explore its therapeutic potential.

### 4.3 miR-135a and Target Genes

MiR-135a, a member of the miRNA family, has been found to play a significant role in the development of various neoplastic and non-neoplastic conditions, including brain damage. The *MYC* proto-oncogene is a crucial regulator of cellular processes, and its dysregulation has been implicated in brain tumors and other disorders. Studies indicate that miR-135a can modulate the expression of *MYC* in brain cells. Specifically, miR-135a has been reported to down-regulate the expression of *MYC* by targeting determinate signaling pathways involved in its regulation, such as the TRAF5/AKT/β-catenin pathway. By inhibiting this pathway, miR-135a suppresses the expression of cyclin D1 and c-MYC, two key proteins involved in cell cycle regulation and cell proliferation [73]. The dysregulation of *MYC* has been associated with brain damage, including malignant primary brain tumors such as glioblastoma, which pose significant treatment challenges and carry poor prognoses. Understanding the potential interactions between miR-135a and *MYC* in the context of brain damage could provide insights into the underlying mechanisms and potential therapeutic targets [74]. However, it is important to acknowledge that the role of miR-135a on the *MYC* gene in brain damage is still not fully understood. Further research is needed to elucidate the specific molecular mechanisms involved and explore the therapeutic potential of targeting miR-135a-MYC interactions in brain damage. Overall, the involvement of miR-135a in regulating cellular processes relevant to brain damage and its potential interaction with the *MYC* gene makes it a promising area of investigation for understanding the underlying mechanisms and developing potential therapeutic strategies.

While miR-135a has been studied in the context of various biological functions and disorders, its specific interaction with the *JAK2* gene in brain damage is not well documented. However, it is worth noting that *JAK2* is a key regulator of several signaling pathways involved in cellular processes, including inflammation, apoptosis, and cell proliferation [75]. Further research is needed to explore the potential relationship between miR-135a and the *JAK2* gene in the context of brain damage. Unraveling the molecular mechanisms underpinning brain damage and identifying potential therapeutic targets is crucial for the development of effective treatments for neurological disorders.

### 4.4 miR-200c and Target Genes

miR-200c, a member of the miR-200 family, has been found to play a significant role in the regulation of the Zinc Finger E-Box Binding Homeobox 1 (*ZEB1*) gene. The *ZEB1* gene is known to be involved in EMT, a process that is crucial for various physiological and pathological events, including cancer metastasis and tissue repair. Studies have shown that miR-200c directly targets the 3' UTR of *ZEB1* mRNA, leading to its downregulation [76]. This downregulation of *ZEB1* by miR-200c has been linked with the inhibition of EMT and the maintenance of epithelial characteristics across diverse cell types. In the context of brain damage, where EMT-related processes can exacerbate neuronal injury, the role of miR-200c on *ZEB1* becomes particularly relevant [77]. By targeting and downregulating *ZEB1*, miR-200c may contribute to the inhibition of EMT and the preservation of the epithelial phenotype in the brain. Furthermore, the dysregulation of miR-200c-ZEB1 axis has been implicated in various neurological disorders, including brain tumors and neurodegenerative diseases. Altered expression levels of miR-200c and *ZEB1* have been observed in these conditions, suggesting their involvement in disease progression and pathological mechanisms [78]. However, it is important to note that the role of miR-200c, specifically on the *ZEB1* gene in the context of brain damage, is not extensively documented in the available literature. Further research is warranted to fully understand the mechanisms and implications of miR-200c-ZEB1 interactions in brain damage and explore their potential as therapeutic targets.

miR-200c has been found to play a crucial role in regulating the expression of the Tubulin Beta 3 Class III (T*UBB3*) gene in various biological processes, including brain damage. *TUBB3* is a gene associated with congenital fibrosis of extraocular muscles (CFEOM3A), a disorder affecting the growth and guidance of ocular motor nerves. Research suggests that miR-200c exerts a negative regulatory effect on *TUBB3* expression, with dysregulation of the miR-200c-TUBB3 axis being implicated in the progression of various neurological disorders [79]. Notably, research has demonstrated that overexpression of miR-200c targeted *TUBB3* and restored the expression of E-cadherin, a protein essential for maintaining cell adhesion and epithelial phenotype. This indicates that miR-200c may inhibit the process of EMT, preserving the epithelial phenotype in the brain [80, 81]. However, the specific role of miR-200c on the *TUBB3* gene Nevertheless, the precise impact of miR-200c on the *TUBB3* gene in the context of brain damage lacks comprehensive documentation. Additional research efforts are imperative to capture the mechanisms and ramifications of their interactions. Understanding its precise role in regulating *TUBB3* and other genes implicated in neurological disorders holds the potential to ensure valuable insights pertinent to the formulation of innovative therapeutic approaches in the future.

Recent literature has sparked interest in exploring the role of miR-200c on the *BMI1* gene in brain damage. miR-200c, a pivotal miRNA involved in gene regulation, has been found to inhibit EMT by targeting the *BMI1* gene *via* the phospho-AKT pathway [82]. EMT is a process involved in various biological processes, including tissue development, wound healing, and cancer progression. Dysregulation of EMT has also been implicated in brain damage. The miR-200c-mediated inhibition of EMT through targeting the *BMI1* gene suggests a potential therapeutic target for brain damage. Furthermore, post-stroke increases in miR-200c have been shown to contribute to brain cell death, indicating the involvement of miR-200c in brain damage processes. The direct mediation of *BMI1* by miR-200c suggests that *BMI1* is involved in miR-200c functions. *BMI1* is a gene critical for self-renewal in many types of stem cells. Its suppression by miR-200c may have implications for brain damage, as stem cells are involved in the repair and regeneration of damaged brain tissue [83]. Overall, the role of miR-200c on the *BMI1* gene in brain damage is complex and multifaceted. Further research is needed to fully understand the mechanisms and implications of their interactions. However, these findings highlight the potential of miR-200c as a therapeutic target for brain damage and provide insights into the regulation of EMT and stem cell functions in the brain.

In the realm of brain injury, miR-200c has been found to interact with the Zinc Finger E-Box Binding Homeobox 2 (*ZEB2*), playing a crucial role in modulating the progression and recovery of brain injury. Studies have shown that miR-200c negatively regulates *ZEB2* expression by binding to its mRNA, leading to the suppression of *ZEB2* protein synthesis [84]. This regulation has been observed to influence various processes involved in brain injury, including inflammation, neuroplasticity, and cellular repair. Specifically, the downregulation of *ZEB2* by miR-200c has been associated with reduced neuroinflammation, enhanced neuronal survival, and improved functional recovery following brain injury [85]. Understanding the intricate interaction between miR-200c and *ZEB2* has potential implications for the development of targeted therapeutic interventions to promote brain injury recovery.

MiR-200c, a specific miRNA, has emerged as a key player in regulating diverse processes, including brain injury. Within the context of brain injury, miR-200c has been found to interact with the *FN1* gene. *FN1* is a crucial extracellular matrix protein involved in cell adhesion, migration, and tissue repair. miR-200c may play a role in regulating fibronectin levels in the brain, potentially affecting the composition of the extracellular matrix and the progression of brain injury [86]. Studies have shown that miR-200c can downregulate the expression of *FN1*, leading to impaired cellular adhesion and migration processes required for brain injury and recovery. This interaction suggests that miR-200c may play a significant role in modulating the recovery and repair mechanisms following brain injury by influencing *FN1* expression levels. Further research is needed to fully understand the intricate relationship between miR-200c and *FN1* in the context of brain injury and explore potential therapeutic interventions targeting this interaction [87].

### 4.5 miR-451a and Target Genes

The interaction between miR-451a and the macrophage *MIF* gene plays a crucial role in brain injury. MiR-451a has been found to be upregulated in response to brain injury. It acts as a post-transcriptional regulator, binding to the 3'-UTR of the *MIF* gene's mRNA. This binding inhibits the translation of *MIF*, subsequently leading to a decrease in MIF protein levels. This interaction has significant implications in brain injury as *MIF* is known to exacerbate inflammation and neuronal damage. By downregulating *MIF*, miR-451a exerts a neuroprotective effect, reducing inflammation and promoting neuronal survival [88]. Understanding this interaction offers potential therapeutic targets for mitigating the effects of brain injury.

miR-451a has been found to play a significant role in brain injury by interacting with the *CAB39* gene. Studies have shown that miR-451a is downregulated in gliomas and acts as a tumor suppressor by inhibiting cell growth and inducing apoptosis. In these glioma cells, miR-451a directly targets *CAB39*, leading to the inhibition of the PI3K/AKT signaling pathway [44]. The downregulation of miR-451a in brain injury may disrupt this regulatory mechanism, resulting in the dysregulation of the PI3K/AKT pathway and potentially contributing to the progression of the injury [21]. Further research is needed to fully understand the implications of this interaction and its potential therapeutic applications in brain injury.

Research has identified miR-451a as a regulator of the *IL6R* gene and an activator of the JAK2/STAT3 pathway, which regulates the proliferation and apoptosis of multiple myeloma cells. While there is limited direct evidence of the interaction between miR-451a and *IL6R* in brain injury, it is known that IL-6 is a potent pleiotropic cytokine that regulates cell signaling and inflammation. Given that miR-451a targets *IL6R*, its dysregulation in brain injury may potentially affect the IL-6 signaling pathway and contribute to the progression of the injury [89, 90]. However, further research is needed to fully understand the implications of this interaction and its specific role in brain injury.

### 4.6 Potential Applications and Future Research Lines

Our analysis suggests that dysregulation of miR-34b, miR-135, and miR-451a is associated with brain damage and neuroinflammation. Particularly, miR-34b is associated with the progression of brain damage by its interaction with the *MET*, *MYC*, *NOTCH1*, and *TGFBR2* genes [49-65], miR-135a is implicated in the mechanisms of brain damage *via* the *MYC* gene [73-75], and miR-451a is associated with mechanisms of brain damage *via* its interaction with *CAB39* and *IL6R* genes [88-90]. Analyzing the potential clinical relevance of these findings, we can suggest that the miRNA dysregulation of miR-34b, mir-135, and miR-451a could be related to the first phase of brain damage. Therefore, they could be used as new molecular biomarkers. At the same time, hypothesizing their use as a therapeutic intervention, it is possible to suggest their silencing through anti-miRNA oligonucleotides or miRNA inhibitors, reducing, consequently, their expression. These results are in agreement with previous publications. Thangavelu *et al*. [91] and Musso *et al*. [24] demonstrated that the same miRNAs could be used as biomarkers or therapeutic targets in penetrating ballistic-like brain injury. Robles *et al*. [92] remarked on the potential use of miR-135a as a promising marker in cases of intracerebral hemorrhage. Weisz *et al*. [93] demonstrated the pivotal role of miR-451a in chronic TBI.

Conversely, dysregulation of miR-34c is associated with post-brain damage reparative mechanisms due to the interaction with *E2F3, CDK4*, and *MYC* genes [66-72], and miR-200c dysregulation contributes to tissue repair and regeneration through its interaction with *ZEB1, TUBB3, BMI1, ZEB2*, and *FEN1* genes [77-87]. In this way, their role is associated with neuronal cell repair and reduced neuroinflammation, suggesting their potential role as biomarkers for repair processes. Moreover, it could be thought that by increasing their expression, it may be possible to improve brain tissue repair, improving patient outcomes. The protective role of miR-34c in brain damage has been recently confirmed by Shen *et al*. [94]. Its anti-apoptotic and anti-inflammatory activities are mentioned by Tu and Hu [95]. On the contrary, the role of miR-200c is debatable, considering that it has been described as a mediator of brain damage [86, 96]. Further studies are necessary to clarify its potential applications.

Trying to apply this knowledge to the forensic field, the overexpression of miR-34b, mir-135, and miR-451a could be found in order to verify the presence of brain damage in the identification of the cause of death. Contrariwise, the overexpression of miR-34c and miR-200c could be used to date the time from brain damage correlating miRNA levels with disease severity and recovery outcomes. This aspect could be important in healthcare disputes [4].

To validate these data, it is desirable to perform *in vitro* studies using cell-culture models (*i.e*. using cell lines or primary cultures of neuronal cells to investigate the effects of miRNA dysregulation), co-culture systems (by creating co-culture systems using neurons, astrocytes, and microglia, it is possible to study miRNA-mediated interactions between different cell types during brain damage and repair), organoids (miniature 3D brain-like structures), functional assays (investigate the functional consequences of miRNA dysregulation by assessing cell viability, apoptosis, inflammation markers, and neuronal repair processes). Subsequently, the miRNAs that confirmed promising results could be tested *in vivo* studies through animal models (*i.e*., inducing brain injury and analyzing miRNA expression patterns), transgenic mice (*i.e*., knockout), arriving at biomarker validation, testing miRNA expression in the biological samples from animal models.

## 5 STRENGTHS AND LIMITATIONS

This research study exhibits several strengths. Firstly, it thoroughly explored various tools to optimize *in silico* analysis. Additionally, it delved into multiple facets related to miRNA sequences, including cell functions, associations with transcriptional factors, and potential implications in human diseases. Furthermore, the study investigated gene targets and miRNA functionality. However, it also has certain limitations. While the computational approach used for miRNA function pre-evaluation is valuable, it necessitates validation through *in vivo* experimental studies. Moreover, the findings are contingent upon the specific versions of the employed tools; future updates or new tools may alter the results.

## CONCLUSION

Identifying novel theranomiRNAs for brain damage, with the aim of finding novel molecular markers that are useful for treatment as well as diagnosis, is a challenging task for the scientific community. Even though miRNAs have been used extensively in science to diagnose and predict brain injury, our understanding of this field is still quite limited. Improvements in analytical technology and procedures, together with more thorough processing of sample collections, are essential to fully unlock the potential of miRNAs. We believe that, instead of concentrating on finding new miRNAs, it might be more important to support the results that have already been published by creating large-scale research networks that include experts from industry and a variety of disciplines, including epidemiology, statistics, molecular biology, analytical chemistry, bioinformatics, clinical trial design, and health economics. The application of computational studies to clinical research has become one of the pillars that can be deemed indispensable in a historical context where, on the one hand, new technologies are developed on a daily basis and, on the other, the budget set aside for research is getting smaller. For the purpose of designing well-structured scientific investigations that provide results that are both scientifically legitimate and minimize expenses and inquiry timetables, computational studies are essential. In this work, we investigated the biochemical pathways that could connect brain injury to our collection of miRNAs. In the realm of brain injury, the discovery of theranomiRNAs is crucial, highlighting the necessity of well-planned *in vivo* investigations to gather further data in this demanding area of study.

## Figures and Tables

**Fig. (2):**
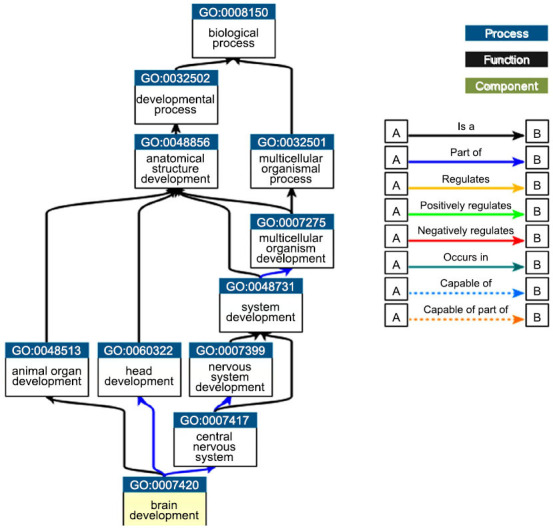
The connection between cell functions and the uploaded miRNA set. These miRNAs are involved in a number of critical processes, including the development of the brain, nervous system, head, and animal organs.

**Fig. (3):**
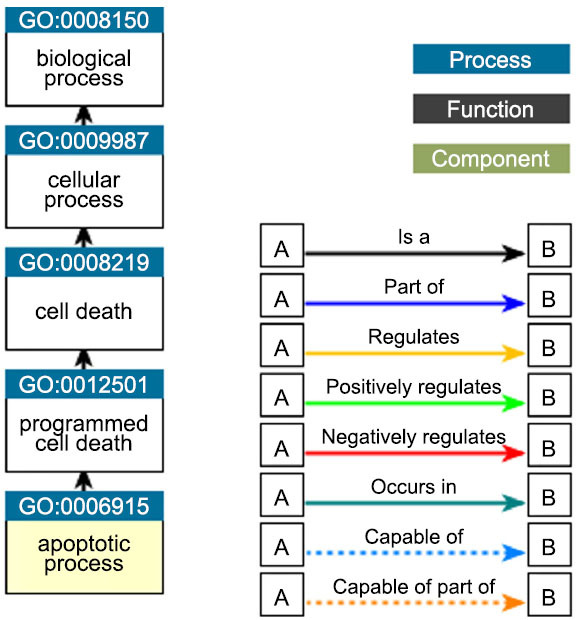
Summary of the GO related to aging processes.

**Fig. (4):**
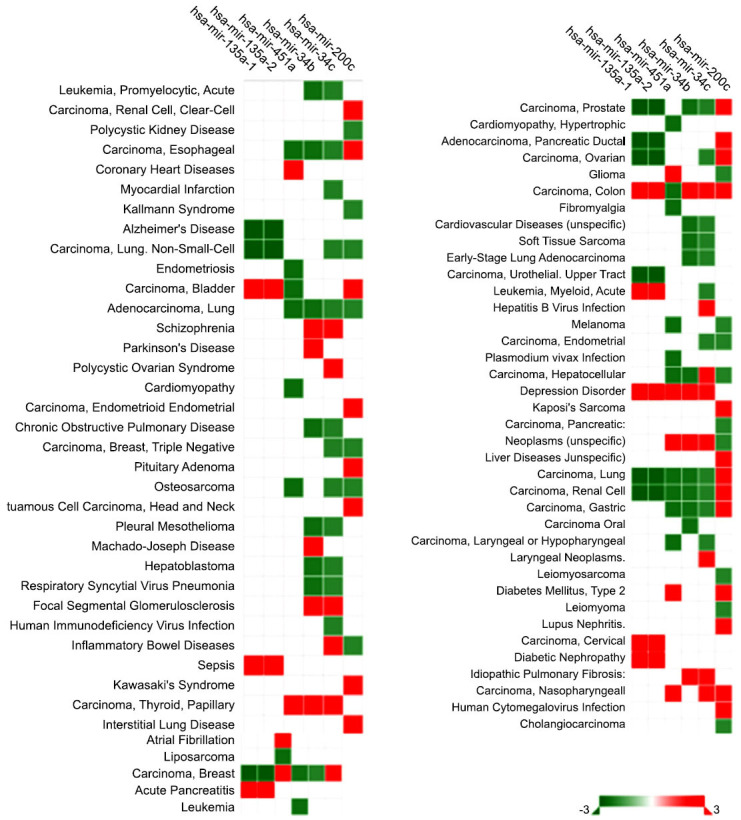
An overview of the primary illnesses associated with this miRNA dataset.

**Fig. (5):**
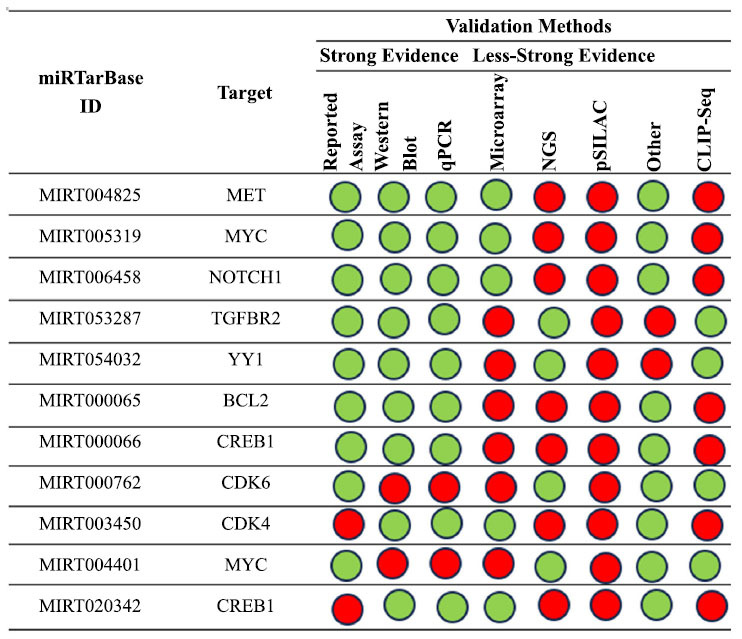
Summary of the miRNA interactions referred to hsa-mir-34b-5p, supported with at least 4 positive tools; green = validated methods, red = unvalidated methods. The methods are distinguished as strong or less strong tools.

**Fig. (7):**
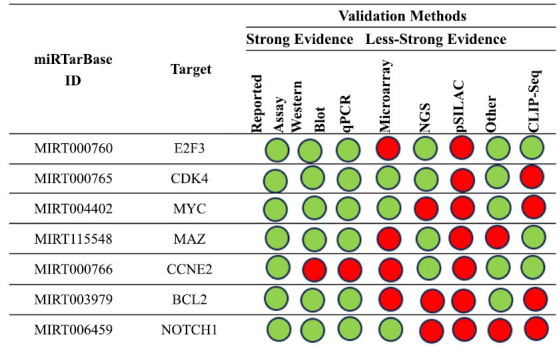
An overview of the miRNA interactions associated with hsa-miR-34c, backed by a minimum of four positive tools; green indicates validated techniques, and red indicates unvalidated methods. The techniques have been classified as either powerful or weaker instruments.

**Fig. (8):**
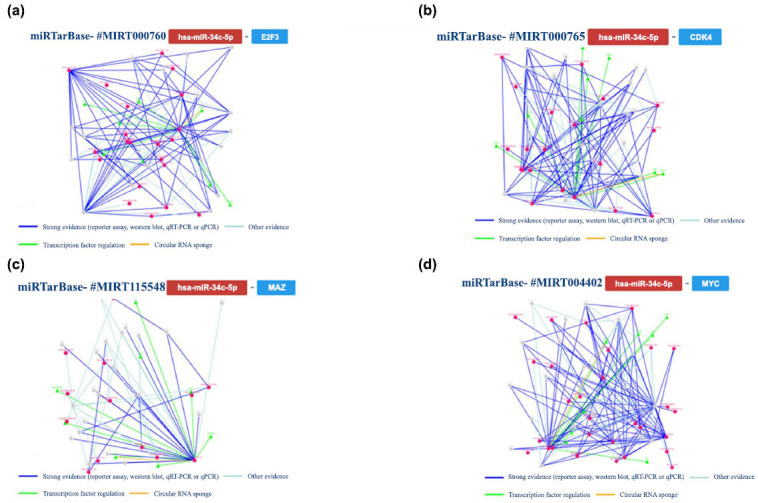
The regulatory network of has-miR-34c and the *E2F3* gene (**a** and **b**) shows the regulatory network of interaction between the tested miRNA and the *CDK4* gene; (**c**) shows the network relative to the *MAZ* and (**d**) *MYC* genes.

**Fig. (9):**
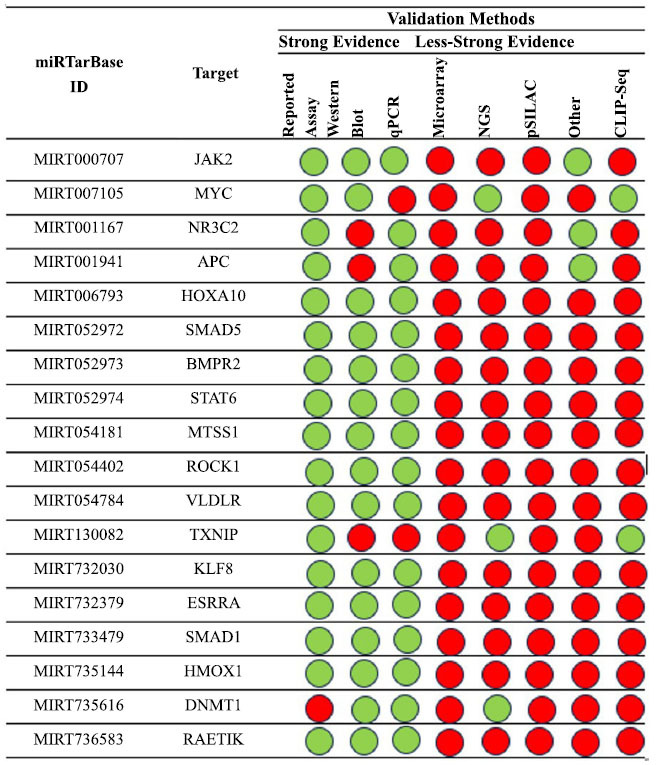
An overview of the miRNA interactions associated with hsa-miR-135a, backed by a minimum of three positive tools; green denotes validated techniques, and red denotes unvalidated methods. The techniques have been classified as either powerful or weaker instruments.

**Fig. (10):**
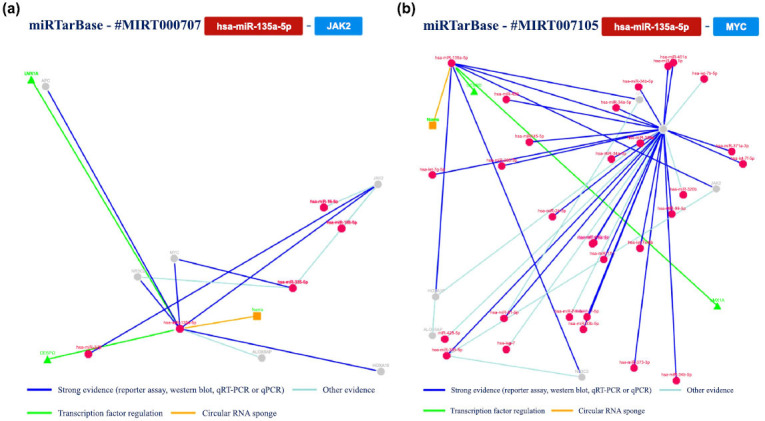
The regulatory network of hsa-miR-135a and *JAK2* and *MYC* gene.

**Fig. (11):**
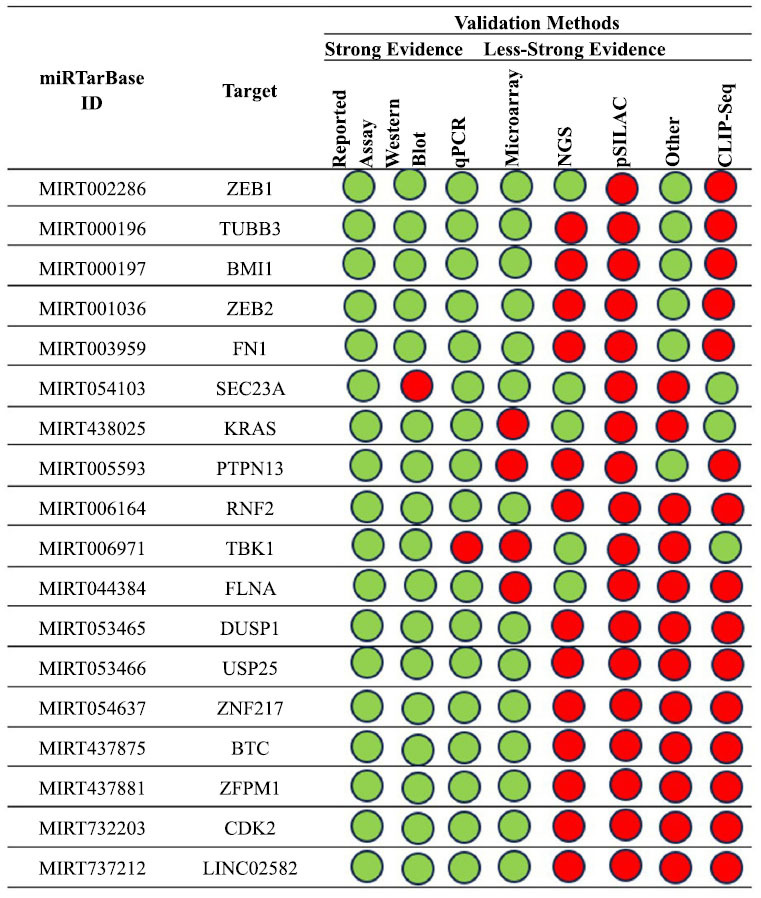
A summary of the hsa-miR-200c interactions that are supported by at least four positive approaches; green indicates validated methods and red indicates unvalidated methods. The techniques have been classified as either powerful or weaker instruments.

**Fig. (12):**
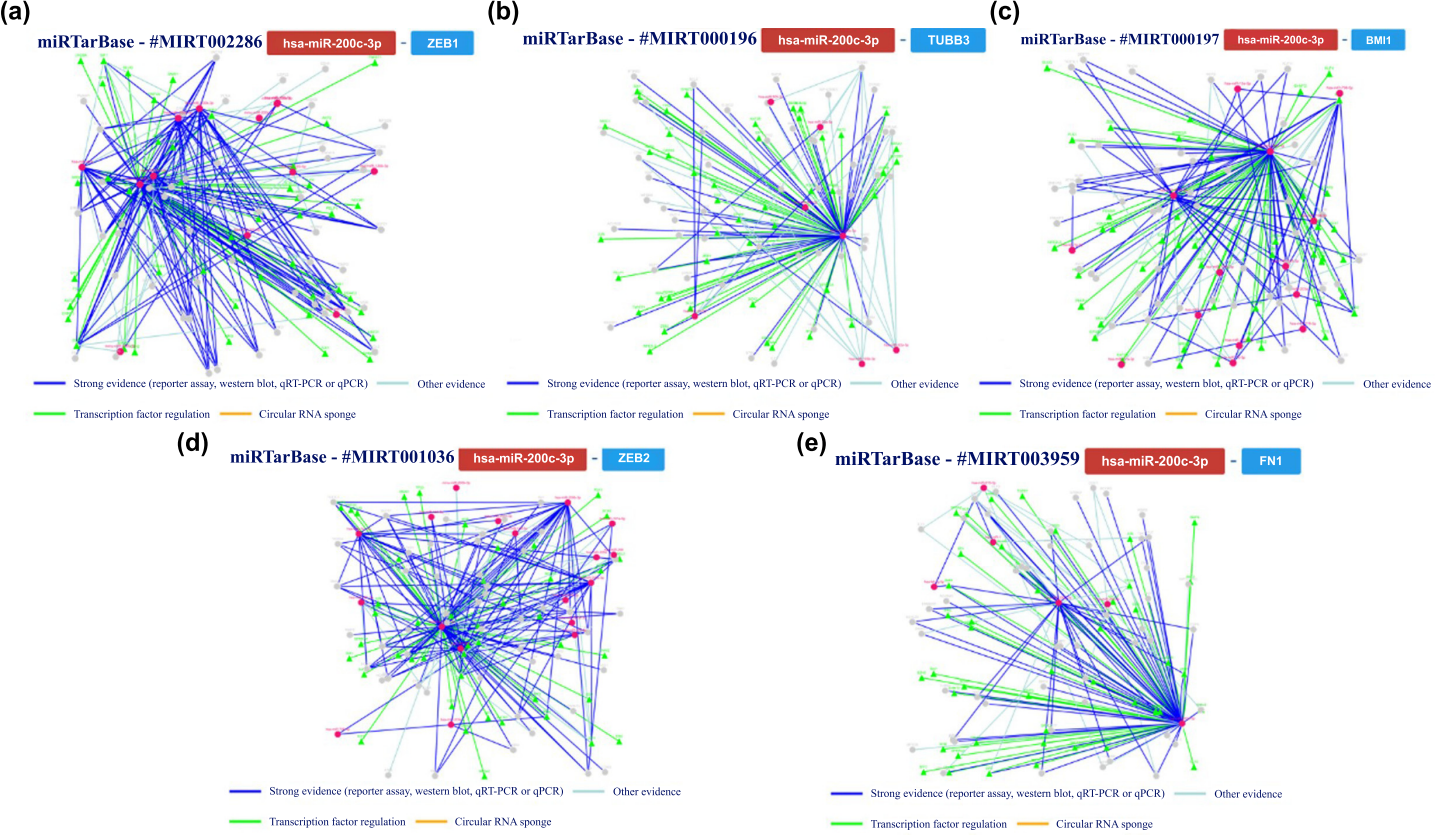
The regulatory network of has-miR-200c and the *ZEB1* gene (**a** and **b**) shows the regulatory network of the interaction between the tested miRNA and the *TUBB3* gene; (**c**) shows the network relative to the *BMI1* gene, *ZEB2* gene (**d**) and *FN1* gene (**e**).

**Fig. (13):**
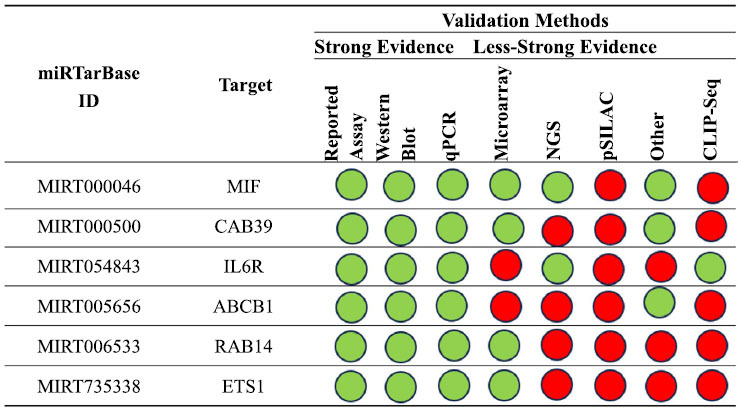
The miRNA interactions with hsa-miR-451a are summarized, backed by a minimum of four positive tools. Green indicates validated techniques, while red indicates unvalidated methods. The techniques have been classified as either powerful or weaker instruments.

**Fig. (14):**
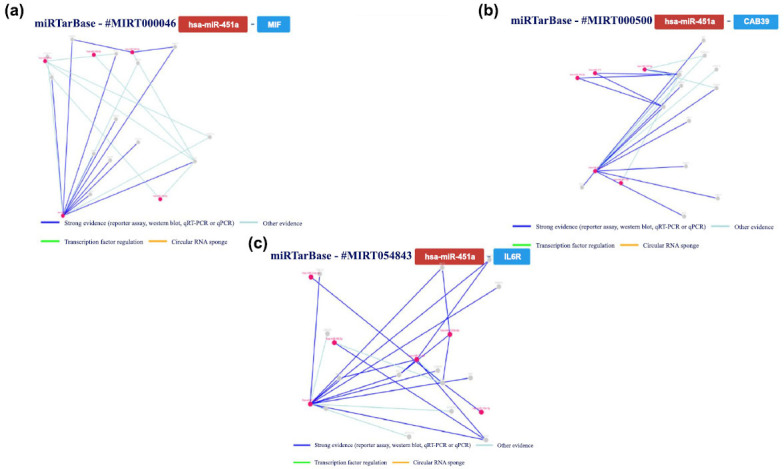
The regulatory network of has-miR-451a and the gene *MIF* (**a** and **b**) the regulatory network of interaction between the tested miRNA and the *CAB39* gene and *IL6R* gene (**c**).

**Table 1:** An overview of the key data for the tested miRNAs, the accession number entered into the tool, the mature miRNA's sequence, and its genomic location.

**miRNA (Mature Sequence)**	**Accession Number**	**Sequence**	**Genomic Localization**
hsa-mir-34b	MI0000742	UAGGCAGUGUCAUUAGCUGAUUG	chr11: 111512938-111513021
hsa-mir-34c	MI0000743	AGGCAGUGUAGUUAGCUGAUUGC	chr11: 111513439-111513515 [+]
hsa-miR-135a	MI0000452	UAUGGCUUUUUAUUCCUAUGUGA	chr3: 52294219-52294308 [-]
hsa-mir-200c	MI0000650	CGUCUUACCCAGCAGUGUUUGG	chr12: 6963699-6963766 [+]
hsa-mir-451a	MI0001729	AAACCGUUACCAUUACUGAGUU	chr17: 28861369-28861440 [-]

**Table 2:** Summary of the main information of the tested miRNAs, the accession number inserted into this tool, the sequence of the mature miRNA, and the genomic localization.

**-**	**Rank, First Authors, Year**	**Article Title**	**No. of ** **Sentences**	**Other Human miRNAs**
hsa-mir-34b	n° 18, Maugeri *et al.*, 2016 [27]	Altered expression of miRNAs and methylation of their promoters are correlated in neuroblastoma.	93	miR-29a-3p, miR-181c-5p and miR-517a-3p
	n° 23, Y. Saito and H. Saito, 2012 [28]	MicroRNAs in cancers and neurodegenerative disorders.	64	miR-9, miR-29
	n° 32, van Rooij and Kauppinen, 2015 [29]	Development of microRNA therapeutics is coming of age.	47	Literature review
	n° 62, Shah *et al.*, 2018 [30]	Regulation of MicroRNAs-Mediated Autophagic Flux: A New Regulatory Avenue for Neurodegenerative Diseases With Focus on Prion Diseases.	22	Literature review
	n° 69, Aranha *et al.*, 2011 [31]	miR-34a regulates mouse neural stem cell differentiation.	20	miR-34a
hsa-mir-34c	n° 28 Hai Yang Hu *et al.*, 2011 [32]	MicroRNA expression and regulation in human, chimpanzee, and macaque brains.	56	/
	n° 33, Rooij and Kauppinen, 2014 [29]	Development of microRNA therapeutics is coming of age.	34	Literature review
	n° 47, De Antonellis *et al.*, 2011 [33]	MiR-34a targeting of Notch ligand delta-like 1 impairs CD15+/CD133+ tumor-propagating cells and supports neural differentiation in medulloblastoma.	29	/
	n° 62, Burgos *et al.*, 2014 [34]	Profiles of extracellular miRNA in cerebrospinal fluid and serum from patients with AD and PD correlate with disease status and features of pathology.	24	Clinical Trial
	n° 85, Saito and Saito, 2012 [28]	MicroRNAs in cancers and neurodegenerative disorders.	17	miR-9, miR-29
hsa-miR-135a-1	n° 7, Podolska *et al.*, 2011 [35]	MicroRNA expression profiling of the porcine developing brain.	27	miR-17, miR-18a, miR-29c, miR-106a, miR-135a and b, miR-221 and miR-222
	n° 18, Zhao *et al.*, 2014 [36]	mRNA-Seq and microRNA-Seq whole-transcriptome analyses of rhesus monkey embryonic stem cell neural differentiation revealed the potential regulators of rosette neural stem cells.	12	let-7 miRNA
	n°41, Garg *et al.*, 2015 [37]	MicroRNA Regulation of Brain Tumour Initiating Cells in Central Nervous System Tumours.	8	Literature review
	n° 34, Smith *et al.*, 2010 [38]	Large-scale expression analysis reveals distinct microRNA profiles at different stages of human neurodevelopment.	9	pred-MIR191, pred-MIR222
	n° 99, Stumpfova *et al.*, 2014 [39]	MicroRNA profiling of activated and tolerogenic human dendritic cells.	3	miR-7, miR-9, miR-155, miR-182, miR-17, miR-133b, miR-203, miR-10a, miR-203, miR-210, miR-30a, miR-449b, miR-134, miR-145, miR-149
hsa-mir-200c	n° 159, Fuschi *et al.*, 2017 [40]	Central role of the p53 pathway in the noncoding-RNA response to oxidative stress.	20	miR-192-5p
	n° 164, Shah *et al.*, 2018 [30]	Regulation of MicroRNAs-Mediated Autophagic Flux: A New Regulatory Avenue for Neurodegenerative Diseases with Focus on Prion Diseases.	19	miRNA-124a-3p, miRNA-136-5p and miRNA-376a-3p miRNA-146a-5p, miRNA-142-3p, miRNA-143-3p, miRNA-145a-5p, miRNA-451a, miRNA-let-7b, miRNA-320, and miRNA-150-5p
	n° 171, Chatterjee *et al.*, 2014 [41]	Studying the system-level involvement of microRNAs in Parkinson's disease.	18	hsa-miR-29a, hsa-miR-9, hsa-let-7a, hsa-let-7i, hsa-miR-19b
	n° 175, Saugstad, 2015 [42]	Non-Coding RNAs in Stroke and Neuroprotection.	16	Literature rewiew
-	n° 209, Meza-Sosa *et al.*, 2014 [43]	microRNAs: key triggers of neuronal cell fate.	13	miRNAs let-7, miRNA-124, miRNA-9, miRNA-134, miRNA-25, miRNA-137
hsa-mir-451a	n° 4, Tian *et al.*, 2012 [44]	miR-451 downregulates the PI3K/AKT pathway through CAB39 in human glioma.	168	/
	n° 24, Bhomia *et al.*, 2016 [45]	A Panel of Serum miRNA Biomarkers for the Diagnosis of Severe to Mild Traumatic Brain Injury in Humans.	30	miR-151-5p, miR-195, miR-20a, miR-328, miR-362-3p, miR-30d, miR-451, miR-486, miR-505, miR-92a
	n° 98, Ren *et al.*, 2010 [46]	MicroRNA-21 inhibitor sensitizes human glioblastoma cells U251 (PTEN-mutant) and LN229 (PTEN-wild type) to taxo1.	9	miR-21
	n° 109, Maes *et al.*, 2009 [47]	MicroRNA: Implications for AD and other Human CNS Disorders.	8	miR-132, miR-124a, miR-133b, miR-9, miR-125b, miR-128, miR-15, miR-146b, miR-181c
	n° 152, Mckiernan *et al.*, 2012 [48]	Reduced mature microRNA levels in association with dicer loss in human temporal lobe epilepsy with hippocampal sclerosis.	4	miR-26a, miR-125b, miR-29a

## Data Availability

The authors confirm that the data supporting the findings of this research are available within the article.

## LIST OF ABBREVIATIONS

AD: Alzheimer's Disease
ALS: Amyotrophic Lateral Sclerosis
APP: Amyloid Precursor Protein
CAB39: Calcium Binding Protein 39
CSCs: Cancer Stem Cells
EGF: Epidermal Growth Factor-like
EMT: Epithelial-mesenchymal Transition
FN1: Fibronectin 1
IL6: Interleukin 6
MIF: Macrophage Migration Inhibitory Factor
miRNAs: microRNAs
mRNA: messenger RNA
PD: Parkinson's Disease
pRB: Retinoblastoma Protein
SVZ: Subventricular Zone
TBI: Traumatic Brain Injury
TGFBR2: Transforming Growth Factor Beta Receptor 2
TUBB3: Tubulin Beta 3 Class III
UTRs: Untranslated Regions
ZEB1: Zinc Finger E-Box Binding Homeobox 1
ZEB2: Zinc Finger E-Box Binding Homeobox 2
